# A Novel Strategy for the Treatment of Aneurysms: Inhibition of MMP-9 Activity through the Delivery of TIMP-1 Encoding Synthetic mRNA into Arteries

**DOI:** 10.3390/ijms25126599

**Published:** 2024-06-15

**Authors:** Sonia Golombek, Isabelle Doll, Louisa Kaufmann, Mario Lescan, Christian Schlensak, Meltem Avci-Adali

**Affiliations:** Department of Thoracic and Cardiovascular Surgery, University Hospital Tuebingen, Calwerstraße 7/1, 72076 Tuebingen, Germany

**Keywords:** aneurysm, synthetic mRNA, cardiovascular diseases, TIMP-1, protein replacement therapies, cardiovascular interventions

## Abstract

Aneurysms pose life-threatening risks due to the dilatation of the arteries and carry a high risk of rupture. Despite continuous research efforts, there are still no satisfactory or clinically effective pharmaceutical treatments for this condition. Accelerated inflammatory processes during aneurysm development lead to increased levels of matrix metalloproteinases (MMPs) and destabilization of the vessel wall through the degradation of the structural components of the extracellular matrix (ECM), mainly collagen and elastin. Tissue inhibitors of metalloproteinases (TIMPs) directly regulate MMP activity and consequently inhibit ECM proteolysis. In this work, the synthesis of TIMP-1 protein was increased by the exogenous delivery of synthetic TIMP-1 encoding mRNA into aortic vessel tissue in an attempt to inhibit MMP-9. In vitro, TIMP-1 mRNA transfection resulted in significantly increased TIMP-1 protein expression in various cells. The functionality of the expressed protein was evaluated in an appropriate ex vivo aortic vessel model. Decreased MMP-9 activity was detected using in situ zymography 24 h and 48 h post microinjection of 5 µg TIMP-1 mRNA into the aortic vessel wall. These results suggest that TIMP-1 mRNA administration is a promising approach for the treatment of aneurysms.

## 1. Introduction

Cardiovascular diseases (CVDs) are the leading cause of death worldwide and cause more than 1.8 million deaths each year, representing 37% of all deaths in the European Union (EU) [1] and placing a significant burden on medical healthcare systems. A subfamily of CVDs are aneurysms, which are caused by progressive focal abnormal dilatation of the arteries due to local weakening of the blood vessel wall. They can develop in various locations throughout the body, including the aorta (abdominal or thoracic aortic aneurysm), blood vessels supplying the brain (cranial aneurysm), and other parts of the body (peripheral aneurysm). 

Aortic aneurysms affect the body’s main artery, the aorta, and if left untreated, can lead to catastrophic dissections and ruptures with massive internal bleeding, with an overall mortality rate of 90% [2,3], corresponding to more than 200,000 deaths per year worldwide [4]. Abdominal aortic aneurysms (AAA) are the most commonly identified aortic aneurysms and have the highest prevalence in the over-65 age group (approximately 4–7% of men and 1–2% of women) [5]. 

Current therapeutic options for the treatment of aneurysms include medical stabilization, monitoring of progression, and surgical treatment for late-stage AAA patients. Medical stabilization relies on regular and systemic administration of antihypertensive drugs to reduce the shear forces on the vessel wall to slow the growth of the aneurysm without being able to restore the fragile vessel. Several drugs, such as β-blockers, calcium antagonists, and angiotensin-converting enzyme (ACE) inhibitors, have been tested in this regard, with some beneficial effects reported but not consistently confirmed in larger and controlled studies. Thus, there is currently no effective and specific drug therapy to stabilize or heal aneurysms. Consequently, the monitoring of aneurysm progression and surgical treatment of patients involving open aneurysm repair (OAR) or endovascular aneurysm repair (EVAR) in late aneurysm stages are the only options to mitigate the risk of fatal rupture [6]. 

Several factors may play a role in the development of AAA, including advanced age, male gender, smoking, family history, hypertension, and atherosclerosis. At the tissue level, extracellular matrix (ECM) degradation leads to aortic wall weakening and dilatation, which may subsequently result in aneurysm rupture with high mortality and morbidity. 

The wall of the arteries consists of three layers: The innermost layer is the tunica intima, which is in contact with the blood and consists of an endothelial cell layer. The second layer, the tunica media, contains smooth muscle cells (SMCs) and the ECM composed of elastic and collagen fibers, which provide stability as well as elasticity. The tunica adventitia is the outer layer composed of fibroblasts, vasa vasorum, lymphatic vessels, nerves, and immune and stem cells [7]. In pathological conditions, such as aortic aneurysms, the vessel wall is destabilized due to defective or deficient structural components. Inflammation and upregulation of proteolytic processes lead to the degradation of the major structural proteins, elastin and collagen, in the aortic vessel wall. The ECM protein elastin is the load-bearing component and is responsible for the elasticity and resilience of large arteries, whereas collagen is responsible for the structural integrity and tensile strength bearing load at high pressures or when elastin fails. Over time, elastin degradation, cyclic strain, and increased wall tension lead to progressive aortic dilatation [8]. 

High levels of elastin degradation products have been found in serum samples from AAA patients [9]. These products might lead to disease progression by increasing chemotaxis and activation of mononuclear cells. Oxidative stress, inflammation, and accelerated SMC apoptosis further promote aortic wall destabilization [8,9,10]. Moreover, inflammation in the vessel wall caused by chemokines and elastin degradation products, among others, leads to the recruitment of leukocytes to the aortic vessel wall and activation of macrophages, which in turn produce pro-inflammatory molecules and matrix metalloproteinases (MMPs) [11,12,13]. 

MMPs are a group of enzymes involved in the physiological turnover of the ECM during wound healing or tissue remodeling processes by degrading structural components, such as elastin or collagen. MMP-2, -9, and -12 play a major role in the degradation of the ECM in degenerative AAAs [12]. In particular, MMP-9, also called gelatinase B, is responsible for the degradation of elastin and collagen type I and IV. Increased MMP-9 plasma levels as well as mRNA expression levels could be detected in AAA compared to healthy aortic vessels [14]. Interestingly, an AAA size dependency of the MMP-9 content was observed in patients with AAA [15]. MMP-9 mRNA expression levels were significantly higher in moderate diameter sized (5- to 6.9 cm) compared to small (<4.0 cm) and large (>7.0 cm) AAAs, indicating a crucial role of MMP-9 in the progression of the AAA process. The activity of MMPs is regulated by their endogenous inhibitors, the family of tissue inhibitors of metalloproteinases (TIMPs), and thus, they also play an important role in ECM remodeling and disease development. Therefore, an imbalance between MMPs and TIMPs has been associated with the pathophysiology and progression of various diseases, such as CVDs [16]. The stoichiometric ratio between the non-covalent binding of TIMP and MMP has been reported to be 1:1 [17]. Thus, maintaining the MMP:TIMP ratio is the principal basis for the development of several new drugs. TIMP-1 is a soluble protein secreted by cells into the ECM and is the endogenous inhibitor of MMP-9 [18]. Moreover, TIMP-1 has a high affinity for almost all active MMPs and can bind to pro-MMP-9, slowing its activation [18]. Decreased TIMP-1 levels have been detected in aortic aneurysms, providing a favorable environment for ECM degradation and further aneurysm expansion [19]. 

A promising new approach for tissue regeneration by reproducing missing or damaged proteins is the use of synthetic messenger RNA (mRNA) [20]. Synthetic mRNA offers the possibility of protein synthesis under physiological conditions. There are already numerous in vivo studies of synthetic mRNA-based drugs for protein replacement therapy, immunotherapy, and vaccine production. The breakthrough came with mRNA-based SARS-CoV-2 vaccines developed during the COVID-19 pandemic, as the first-ever approved mRNA therapeutics [21,22]. For more than two decades, mRNA technology has been optimized. Special attention has been paid to the optimization of mRNA to reduce unwanted immune-activating side effects and to increase mRNA stability and protein expression efficiency [23]. Both can be achieved by incorporating modified nucleotides [24], integrating a 3′poly(A)-tail and a 5′-end cap structure [25], as well as optimization of the codon sequences and untranslated regions [26], and optimizing the codon sequences (increasing GC-rich codons and reducing AU-rich codons) can be used instead of modified nucleotides [27]. Moreover, compared to viral vectors and recombinant proteins, in vitro transcribed mRNA has several advantages, e.g., there is no risk of insertional mutagenesis [28,29], and the protein of interest is only transiently expressed, avoiding potential protein-dependent side effects [28]. The local overexpression of TIMP-1 protein prevented elastin depletion and aneurysm formation as well as rupture in rats [30]. 

In this study, TIMP-1 protein expression was analyzed after the in vitro TIMP-1 mRNA transfection of cells. Afterward, a microinjection-based method was developed to deliver the TIMP-1 mRNA into the vessel wall. The successful expression of TIMP-1 after the delivery of synthetic TIMP-1 mRNA in ex vivo porcine and human aortic vessel walls was demonstrated and the functionality of the produced TIMP-1 protein was evaluated using in situ zymography.

## 2. Results

### 2.1. Transfection of Cells with Synthetic TIMP-1 mRNA

**Figure 1:**
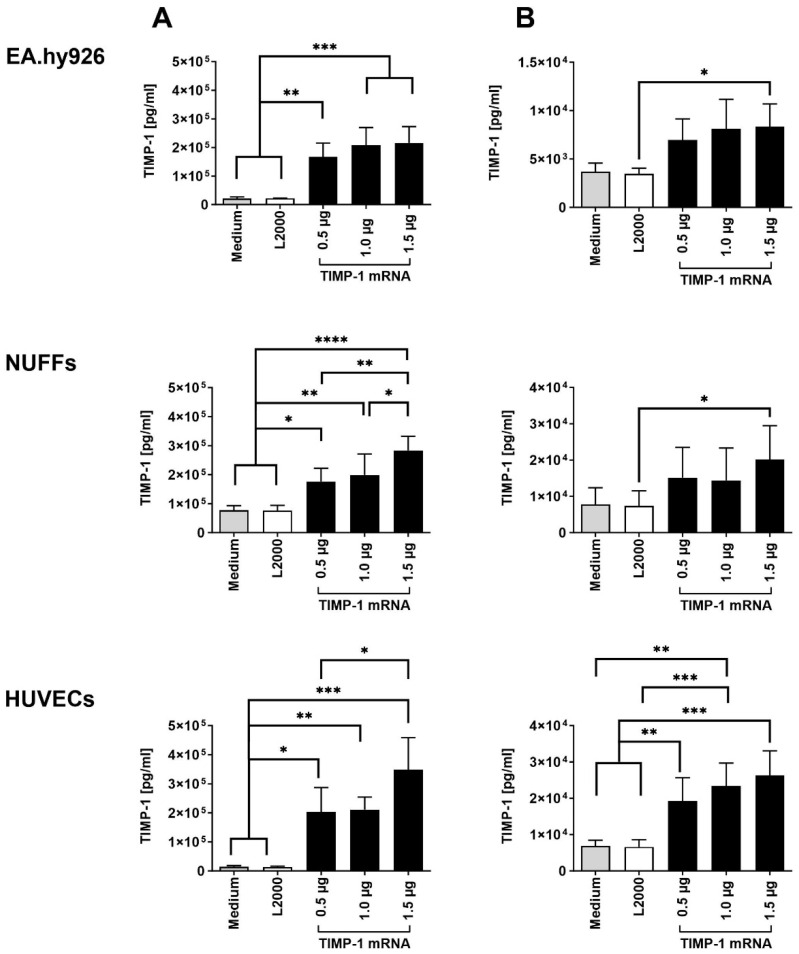
Analysis of TIMP-1 protein expression after the transfection of different cells with synthetic TIMP-1 mRNA. During the analysis, 3 × 10^5^ EA.hy926 cells, NUFFs, or HUVECs were transfected with 0.5, 1.0, and 1.5 µg TIMP-1 mRNA complexed 1:1 with Lipofectamine 2000 (L2000). After 4 h incubation at 37 °C and 5% CO_2_, the transfection complexes were replaced with the appropriate corresponding cell culture medium. After additional incubation for 24 h at 37 °C and 5% CO_2_, the TIMP-1 protein concentration in the (**A**) supernatants and (**B**) cell lysates was determined using human TIMP-1 specific ELISA. Cells treated with medium or medium containing L2000 served as controls. The results are shown as the mean + SD (n = 3). Statistical differences were determined using one-way ANOVA followed by Tukey’s multiple comparisons test. (* *p* < 0.05, ** *p* < 0.01, *** *p* < 0.001, and **** *p* < 0.0001).

To analyze TIMP-1 protein production, 3 × 10^5^ EA.hy926 cells, NUFFs (newborn foreskin fibroblasts), or HUVECs (human umbilical vein endothelial cells) were transfected with 0.5, 1.0, or 1.5 µg synthetic TIMP-1 mRNA. Using ELISA, significantly increased TIMP-1 levels were detected in the supernatants of all cell types 24 h after TIMP-1 mRNA transfection (Figure 1A). Except for EA.hy926 cells, increasing the TIMP-1 mRNA concentration to 1.5 µg resulted in a significant increase in TIMP-1 protein expression. Higher levels of TIMP-1 were detected in the supernatants of the cells than in the cell lysates (Figure 1B), indicating the secretion of TIMP-1 protein into the extracellular space after translation. In EA.hy926 cells and NUFFs, only transfection of 1.5 µg TIMP-1 mRNA resulted in significantly higher TIMP-1 levels in cells compared with cells transfected with transfection reagent (L2000) alone (Figure 1B). In HUVECs, transfection of 0.5 µg TIMP-1 mRNA already resulted in significantly increased TIMP-1 levels in cells. 

### 2.2. Delivery of Synthetic mRNA into the Aortic Vessel Wall by Microinjection

**Figure 2:**
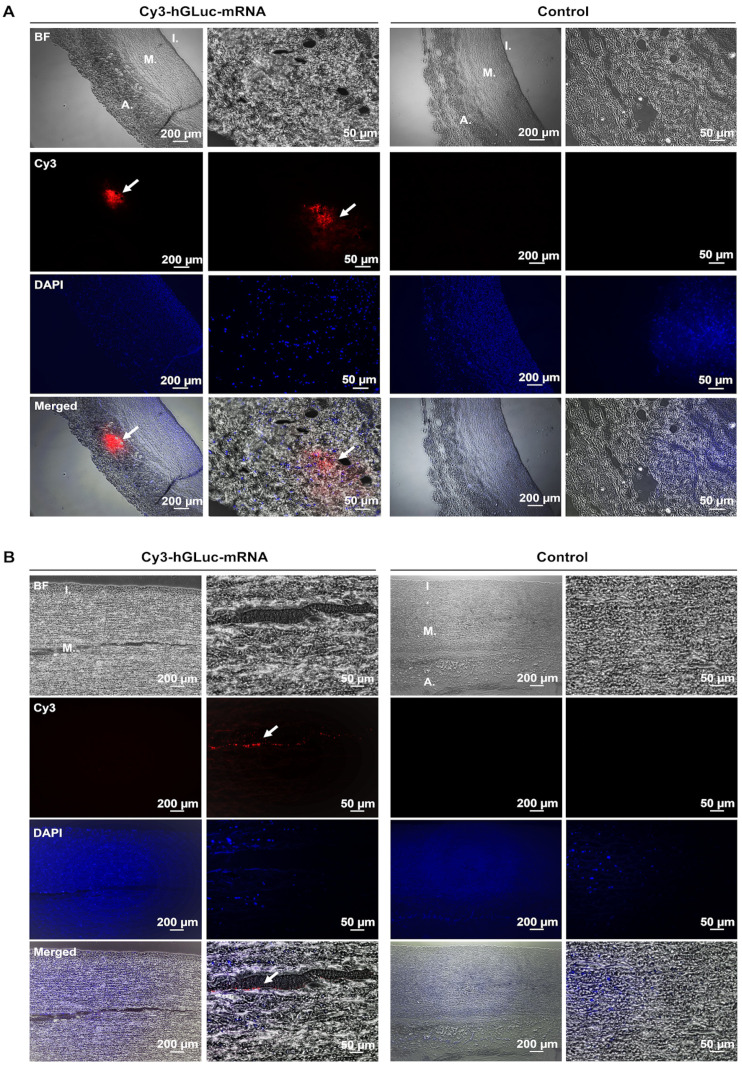
Application of synthetic hGLuc mRNA into ex vivo porcine and human aortic vessel walls. 1 μg of Cy3-labeled hGLuc mRNA complexed with 1 μL L2000 in 50 μL Opti-MEM was injected into porcine (**A**) and human (**B**) aortic vessel walls from the intraluminal side. After microinjection, the samples were fixed in 4% PFA, and paraffin sections were prepared (2.5 μm). Cell nuclei were stained with DAPI. White arrows indicate the Cy3-labeled hGLuc mRNA in the tunica media and adventitia. DAPI (blue), Cy3-labeled hGLuc mRNA (red). I.: tunica intima, M.: tunica media, and A.: tunica adventitia. Left column: 5× magnification, right column: 40× magnification. (porcine n = 3; human n = 4).

To investigate the delivery of synthetic mRNA into the blood vessel wall, 1 µg of Cy3-labeled hGLuc mRNA was injected ex vivo using hollow microneedles from the intraluminal side into porcine and human aortic vessel walls. In paraffin cross-sections, the localization of Cy3-labeled hGLuc mRNA was analyzed using fluorescence microscopy. In these histologic sections, the individual compartments of the vessels, tunica intima, tunica media, and tunica adventitia, were readily recognizable. The cell nuclei were stained with DAPI (blue). Cy3-labeled hGLuc mRNA (red) was detected mainly in the tunica adventitia of the porcine aortic wall (Figure 2A) and the tunica media of the human aortic wall (Figure 2B). 

**Figure 3:**
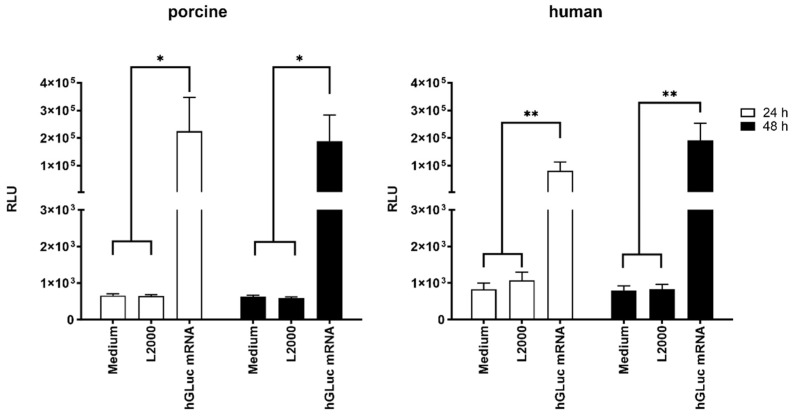
Protein expression after the injection of synthetic hGLuc mRNA into ex vivo porcine and human aortic vessel walls. Luciferase activity was detected after ex vivo microinjection of 1 µg synthetic hGLuc mRNA complexed with 1 µL L2000 in the porcine aorta (**left**) and human aorta (**right**). Cell supernatants were analyzed using luciferase assay after incubation at 37 °C and 5% CO_2_ for 24 and 48 h, respectively. Aortic vessels injected with medium or medium containing 1 µL L2000 were used as the control. For statistical analysis, two-way ANOVA test with subsequent Tukey’s multiple comparisons test was performed (* *p* < 0.05; ** *p* < 0.01). The results are shown as means + SEM (porcine n = 4; human n = 5).

To determine whether the administered hGLuc mRNA could be translated into protein, the supernatants of ex vivo cultivated aortic vessels were collected at 24 and 48 h post-injection. Compared with control groups injected with medium alone or medium with L2000, microinjection of hGLuc mRNA in both human as well as porcine aortic vessels led to significantly increased luciferase activity after 24 h of incubation (Figure 3). After 48 h of incubation, similar luciferase activity was measured in the supernatant of porcine vessels as after 24 h, indicating that most of the injected mRNA was translated in the first 24 h of incubation (Figure 3, left). In human aortic vessels, slightly higher luciferase activity was detected after 48 h compared to 24 h. Although cell activity might be impaired in human pathogenic vessels derived from aortic aneurysms compared with healthy vessels, a significant amount of expressed luciferase protein was detected after 24 and 48 h of incubation after mRNA injection (Figure 3, right).

### 2.3. Production of TIMP-1 Protein after the Delivery of Synthetic TIMP-1 mRNA in the Porcine Aortic Vessel Wall

**Figure 4:**
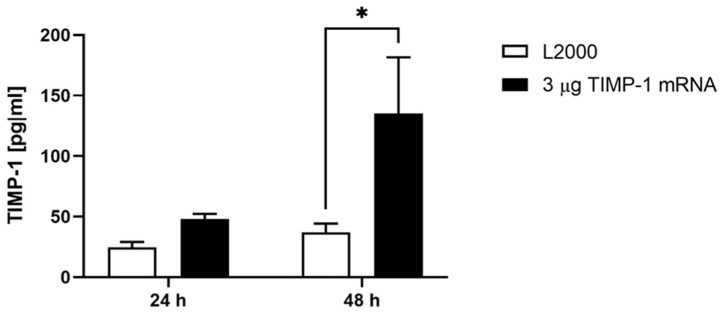
Detection of TIMP-1 levels in the supernatant of porcine aorta after the microinjection of 3 µg TIMP-1 mRNA. 3 μg of TIMP-1 mRNA was complexed with L2000 in 50 μL Opti-MEM and injected into the porcine aortic vessel wall. TIMP-1 levels in the supernatant were detected 24 and 48 h post injection using TIMP-1 ELISA. The results are shown as means + SEM (n = 3). Statistical differences were determined using two-way ANOVA followed by Tukey’s multiple comparisons test. (* *p* < 0.05).

To determine whether the administered TIMP-1 mRNA leads to increased TIMP-1 protein expression, the supernatants of ex vivo cultivated porcine aortic vessels were collected at 24 and 48 h post injection with 3 µg TIMP-1 mRNA. Significantly increased TIMP-1 levels were detected after 48 h of incubation using ELISA (Figure 4).

### 2.4. Detection of MMP-9 Inhibition after the Application of Synthetic TIMP-1 mRNA in Porcine and Human Aortic Vessel Walls

**Figure 5:**
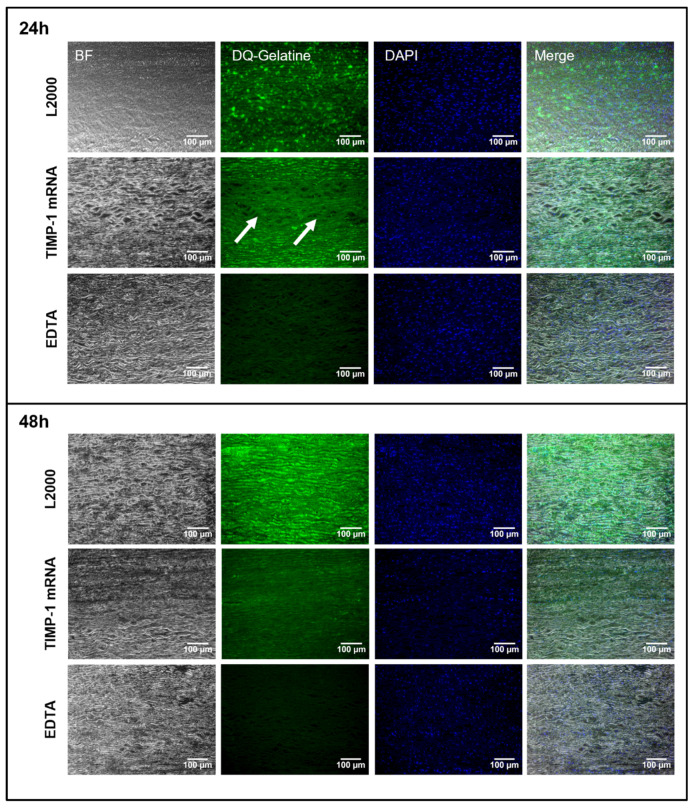
Analysis of MMP activity using in situ zymography in porcine aorta after the microinjection of human TIMP-1 mRNA and incubation for 24 and 48 h. During the analysis, 5 µg of TIMP-1 mRNA complexed 1:1 with L2000 in 50 µL Opti-MEM was injected ex vivo from the side of the intima into the porcine aorta. Tissues were incubated for 24 and 48 h at 37 °C and 5% CO_2_. Injection of L2000 only in Opti-MEM served as a control. Post incubation, the tissues were fixed, and 5 µm paraffin sections were prepared. MMP-9 activity was visualized by using DQ-gelatin-based in situ zymography. To verify enzymatic gelatin cleavage by MMPs, control sections were inhibited with EDTA before the addition of DQ substrate. Cell nuclei were stained using a DAPI-containing mounting medium. DQ gelatin: green; nuclei: blue. 20× magnification; (n = 3). Arrows indicate areas with decreased DQ-gelatine fluorescence signal.

After demonstrating that ex vivo aortic tissue can express the desired protein up to 48 h post injection of synthetic mRNA using hGLuc encoding mRNA, the same ex vivo model was used to evaluate the functionality of expressed TIMP-1 protein. In situ zymography can visualize and assess the proteolytic activity of MMP-9 in histological sections using the fluorescently labeled MMP substrate DQ-gelatin. Enzymatic cleavage of DQ-gelatin by active MMPs results in a green fluorescence signal [31]. Since the activity of MMP-9 is inhibited by EDTA, which binds catalytically necessary Zn^2+^ ions, the tissue sections incubated with EDTA served as negative controls for the in situ zymography. TIMP-1 binds covalently to MMP-9 and inhibits DQ-gelatin cleavage. Thus, the ability of TIMP-1 protein, which is produced after the microinjection of TIMP-1 mRNA into the vessel wall, to inhibit MMP-9 was investigated in both porcine and human aortic vessels. For this purpose, 5 µg of human synthetic TIMP-1 mRNA was injected ex vivo into porcine and human aortic vessels from the side of the intima and incubated for 24 or 48 h. After fixation, MMP-9 activity was assessed in paraffin sections using in situ zymography, MMP-9-dependent DQ-gelatin cleavage efficiency was determined, and TIMP-1 mRNA-treated versus untreated groups were compared. Both 24 and 48 h after injection of TIMP-1 mRNA, a significantly lower signal of DQ-gelatin was detected in the porcine vessel wall than in vessels treated with L2000 alone (Figure 5). As expected, the lowest MMP-9 activity was detected in EDTA-treated vessel sections. The synthetic mRNA could be also microinjected from the side of the adventitia in porcine aortic samples (Figure S1). Here, the results were similar to those obtained with mRNA injections from the side of the intima.

The ECM structures of the porcine (Figure S2) and human (Figure S3) aorta samples were also analyzed after hematoxylin and eosin, elastin, and collagen staining of the histological sections. No differences between the control and treated groups could be observed.

**Figure 6:**
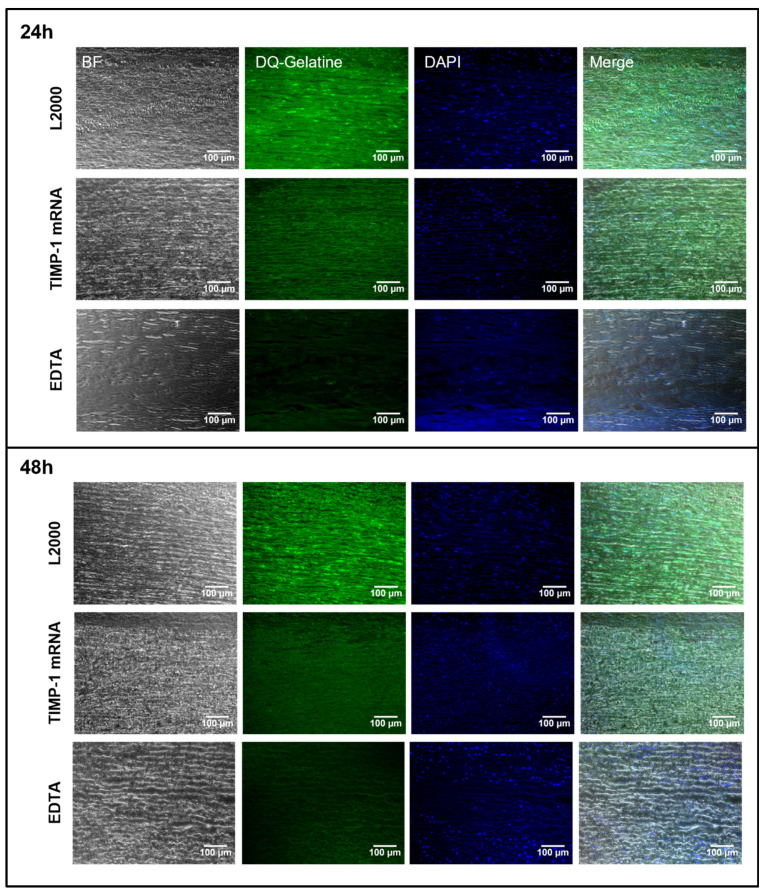
Analysis of MMP activity using in situ zymography in the human aorta after microinjection of human TIMP-1 mRNA and incubation for 24 and 48 h. During the analysis, 5 µg of TIMP-1 mRNA complexed 1:1 with L2000 in 50 µL Opti-MEM was injected ex vivo from the side of the intima into the human aorta. Tissues were incubated for 24 and 48 h at 37 °C and 5% CO_2_. Injection of L2000 only in medium served as a control. Post-incubation tissues were fixed, 5 µm paraffin sections were prepared, and MMP-9 activity was visualized by using DQ-gelatin-based in situ zymography. To verify enzymatic gelatin cleavage by MMPs, control sections were inhibited with EDTA before the addition of DQ substrate. Cell nuclei were stained using a DAPI-containing mounting medium. DQ gelatine: green; nuclei: blue. 20× magnification; (n = 4).

Similar results were obtained in human aortic tissue after the injection of TIMP-1 mRNA and incubation for 24 and 48 h (Figure 6). Here, a strongly reduced DQ-gelatin signal was observed both 24 and 48 h after the injection of synthetic TIMP-1 mRNA compared with the L2000 control groups. Thereby, the inhibitory function of the produced TIMP-1 protein was confirmed. The proteolytic MMP-9 activity was successfully inhibited and decreased in mRNA-treated tissues.

### 2.5. Analysis of TIMP-1 mRNA Translation Efficiency by Variation of Nucleotide Modifications 

**Figure 7:**
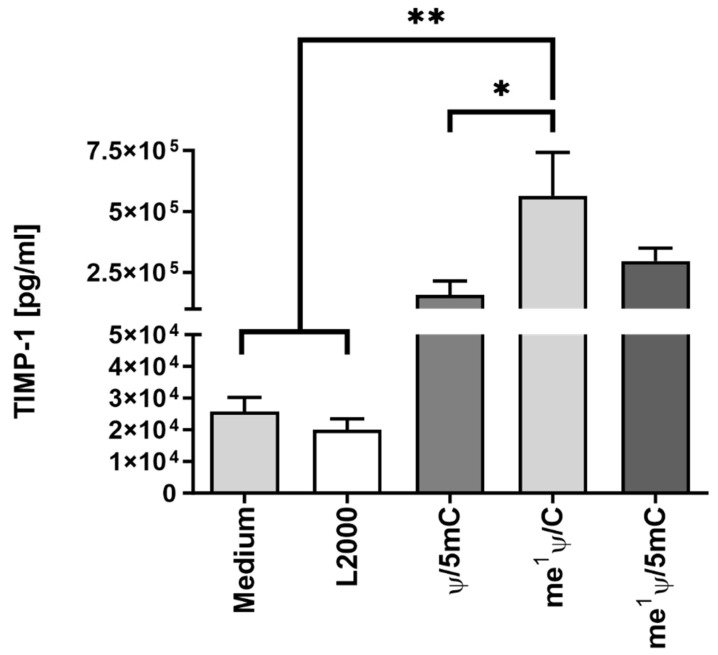
Impact of nucleotide modification on synthetic TIMP-1 mRNA expression efficiency in EA.hy926 cells. First, 3 × 10^5^ EA.hy926 cells were seeded and transfected with 1.5 µg of TIMP-1 mRNA complexed with 1.5 µL of L2000 in Opti-MEM for 4 h at 37 °C and 5% CO_2_. Thereafter, the transfection complexes were replaced by cell culture medium, and the cells were incubated at 37 °C and 5% CO_2_. Cells treated with Opti-MEM or Opti-MEM plus L2000 served as controls. After 24 h, the TIMP-1 concentration was determined in collected supernatants via human TIMP-1-specific ELISA. The results are shown as the mean + SEM (n = 3). Statistical differences between the mRNA-treated groups were determined using one-way ANOVA following Tukey’s multiple comparison test (* *p* < 0.05, ** *p* < 0.01).

To improve the protein expression efficiency of the mRNA, different nucleotide modifications were incorporated into the mRNA and tested. In the prior analysis, the nucleotide modifications Ψ/5mC were used. Ψ was replaced by me^1^Ψ, and TIMP-1 mRNA was modified with either me^1^Ψ/5mC or me^1^Ψ only. Next, 1.5 µg of each mRNA variant was complexed with L2000, and 3 × 10^5^ EA.hy926 cells were transfected. After 24 h, protein translation efficiency was analyzed via TIMP-1-specific ELISA in supernatants (Figure 7). Through the incorporation of me^1^Ψ only, the protein expression efficiency was significantly enhanced. The additional modification with 5mC did not improve translation. 

## 3. Discussion

Increased MMP activity is known to play a significant role in various diseases, including CVDs as well as other diseases like cancer [16]. In cardiovascular diseases, MMPs are involved in processes such as plaque destabilization, vascular remodeling, and aneurysm formation [32]. In cancer, MMPs facilitate tumor invasion, metastasis, and angiogenesis [33]. 

AAAs are characterized by arterial dilatation, degeneration of arterial architecture, disruption of the ECM, inflammatory infiltration, oxidative stress, and, in particular the presence of matrix-degrading enzymes like MMPs, necessitating multifactorial potential treatments. ECM degradation, particularly mediated by matrix MMPs, is considered crucial for AAA occurrence and progression. Due to its multifactorial pathogenesis, potential AAA treatments have included anti-inflammatory agents, endogenous proteinase inhibitors such as TIMPs, and genetic and pharmacological inhibition of MMPs [34]. Early attempts to develop MMP inhibitors were based on hydroxamate-based inhibitors, which were based on the structure of collagen and contained a group that inactivates the catalytic zinc ion via chelation [35]. However, this approach led to intolerable side effects like joint stiffening [36]. Later, beyond others, antibody-based therapeutics were developed for selective inhibition of MMPs [37,38,39]. Multiple studies have proven that the administration of doxycycline suppresses aortic dilation, inhibits MMP-9 expression and activity, and reduces the incidence of AAA formation [40]. However, currently, the antibiotic substance doxycycline is the only MMP inhibitor approved by the Food and Drug Administration (FDA) for use in humans [41] and is used to treat periodontitis [42]. Thus, there is a great need to develop selective and locally effective MMP inhibitors, which are also well tolerated. A very interesting approach is using antibody-conjugated nanoparticles encapsulating different MMP inhibitors such as batimastat, doxycycline, or pentagalloylglucose in AAA models showing positive effects on aneurysms [43,44,45]. In addition, the nanoparticles can specifically target the damaged tissue area following systemic application by specific binding of the conjugated antibody to the damaged elastic fibers present in the affected vessel area.

TIMPs could form the basis for another new class of MMP inhibitors. Studies using MMP- and TIMP-knockout mice have definitively shown the importance of MMP/TIMP imbalances in the onset and progression of AAA [11,46], making the endogenous MMP inhibitor TIMP-1 a relevant candidate. Allaire et al. used retroviral vectors to introduce TIMP-1 cDNA into SMCs, which they then injected into the aortic lumen of rats with aneurysms [30]. Local TIMP-1 overexpression prevented aneurysmal degeneration and rupture. Since MMPs also lead to unstable plaques in atherosclerosis, Rouis et al. injected TIMP-1 DNA via an adenoviral vector venously into an atherosclerotic mouse model [47], which resulted in high hepatic expression of TIMP-1. The increased TIMP-1 concentration in the plasma led to a significantly reduced number of atherosclerotic lesions in atherosclerosis-susceptible hypercholesterolemic apoE−/− mice. While this is a promising strategy, the further effects of a systemic increase in TIMP-1 on the organism still need to be evaluated. Giraud et al. showed that gingival fibroblasts (GFs) with increased secretion of TIMP-1 deposited on the adventitia of AAAs decreased inflammation and ECM destruction and prevented aneurysm progression and rupture [48]. 

mRNA-based therapeutics represent an innovative approach for producing effective proteins directly within the targeted cells and in affected areas. Unlike DNA, synthetic mRNA circumvents the need to enter the cell nucleus, thus mitigating the risk of insertional mutations. Moreover, it also offers the advantage of fast, uncomplicated, and cost-effective production. Additionally, its transient activity and subsequent elimination via physiological degradation pathways curtail possible adverse effects. Notably, existing mRNA therapeutics, such as vaccines, have demonstrated their adaptability to swiftly respond to viral mutations, exemplifying the versatility of this approach [28]. Despite the most common use of synthetic mRNA as vaccines, mRNA-based protein replacement therapeutics have already entered the clinical stage, offering tremendous potential as new therapy options for several diseases [49,50]. Nevertheless, tissue- and cell-specific targeted delivery still remains one of the biggest hurdles.

To our knowledge, we have demonstrated for the first time the applicability of synthetic TIMP-1 mRNA as a new approach for the inhibition of MMP activity in blood vessels. Following these promising results, we plan to conduct in vivo studies in aneurysm models. These will allow a more comprehensive evaluation of the TIMP-1 mRNA effect in a physiologically relevant context. However, the current ex vivo data provide essential insights that will guide our in vivo research. 

In our study, the intraluminal injection of the synthetic TIMP-1 mRNA using microneedles resulted in successful delivery to the tissue and the expression of the target protein, serving as a suitable delivery method for the used ex vivo model. Further possible application methods for therapeutic use are mRNA-coated stents, direct injection into the aortic wall via a catheter, or systemic targeted delivery systems such as nanoparticles. The intraluminal route using a catheter infusion was used in a recent study for the local delivery of pentagalloylglucose to AAAs in pigs. A first-in-human pilot study is being conducted to assess the novel localized treatment for stabilizing small- to medium-sized infrarenal abdominal aortic aneurysms [51].

In summary, the use of synthetic TIMP-1 mRNA represents a promising new strategy for the treatment of cardiovascular diseases characterized by the loss of elastic structural proteins or increased inflammation, such as aneurysms, dissections, and atherosclerosis, by decreasing MMP-9 activity. It is therefore an innovative and novel strategy that has the potential to prevent the progression of aneurysms in the future.

## 4. Materials and Methods

### 4.1. Cultivation of Cells 

EA.hy926 cells (ATCC) and human fibroblasts (NUFFs, newborn foreskin fibroblasts, AMS Biotechnology (Europe) Ltd., Abingdon, UK) were cultivated in Dulbecco’s modified Eagle medium (DMEM) with high glucose and l-glutamine with 10% heat-inactivated fetal bovine serum (FBS) at 37 °C and 5% CO_2_. Cells were washed once with Dulbecco’s phosphate-buffered saline (DPBS) and detached with 0.05% trypsin-EDTA. All cell culture reagents were obtained from Thermo Fisher Scientific (Waltham, MA, USA). Human umbilical vein endothelial cells (HUVECs, (PromoCell, Heidelberg, Germany)) were cultivated in VascuLife EnGS endothelial cell culture medium (without hydrocortisone) (Lifeline Cell Technology, Frederick, MD, USA) at 37 °C and 5% CO_2_. The cells were washed once with DPBS and detached using 0.04% trypsin/0.03% EDTA and trypsin neutralizing solution (TNS) 0.05% in 0.1% BSA (both Promocell, Heidelberg, Germany). Cell culture flasks were coated with 0.1% gelatin (Fluka, Morristown, NJ, USA) in PBS for 15 min at room temperature (RT) before seeding the cells. The medium was changed every 3–4 days, and the cells were passaged upon reaching a confluency of 80–90%.

### 4.2. mRNA Synthesis

Synthetic mRNA was produced via in vitro transcription (IVT) as previously described [52]. Plasmids containing either secretable humanized Gaussian luciferase (hGLuc) or human TIMP-1 encoding sequences were ordered from Eurofins Genetics (Ebersberg, Germany). These plasmids were used to generate the DNA templates containing the coding sequence of hGLuc or TIMP-1 via PCR. Therefore, 50–100 ng of plasmid and the HotStar HiFidelity Polymerase Kit (Qiagen, Hilden, Germany) were used according to the manufacturer’s instructions. Forward (5′-TTGGACCCTCGTACAGAAGCTAATACG-3′) and reverse primers (5′-T120-CTTCCTACTCAGGCTTTATTCAAAGACCA-3) were purchased from ELLA Biotech (Martinsried, Germany). PCR was performed using the following PCR cycling conditions: 94 °C for 3 min, 30 cycles of denaturation to generate single strands at 94 °C for 45 s, hybridization of primers at 60 °C for 1 min, elongation at 72 °C for 1 min, and final elongation at 72 °C for 5 min. The amplified PCR products were purified using the QIAquick PCR purification kit (Qiagen, Hilden, Germany) according to the manufacturer’s instructions. The concentration and purity of the DNA were analyzed photometrically, and the size of the amplified DNA fragments was controlled via 1% agarose gel electrophoresis. 

IVT was performed using a T7 MEGAscript Kit (Life Technologies, Darmstadt, Germany) according to the manufacturer’s instructions. Therefore, 1.5 µg of the hGLuc or TIMP-1 template DNA was used. During IVT, a 2.5 mM 3′-O-Me-m7G(5′)ppp(5′)G RNA cap structure analog (ARCA, New England Biolabs, Frankfurt am Main, Germany) and nucleotides (1.875 mM GTP, 7.5 mM ATP, 7.5 mM 5-methyl-CTP (5mC), 7.5 mM Ψ). were incorporated. This mRNA modification was named Ψ/5mC. To analyze the impact of nucleotide modification on TIMP-1 protein expression, TIMP-1 mRNA was also produced with 7.5 mM N1-methylpseudouridine (me^1^Ψ) instead of Ψ and 7.5 mM 5mC (named me^1^Ψ/5mC) or 7.5 mM CTP (named me^1^Ψ/C). ATP, GTP, and CTP from the MEGAscript T7 Kit were used, and the others were from TriLink (BioTechnologies, San Diego, CA, USA). To each IVT reaction, 40 U RiboLock RNase inhibitor (Thermo Scientific, Waltham, MA, USA) was added, and the IVT reaction was incubated for 4 h at 37 °C. For the removal of the DNA template, 1 µL of TurboDNase was added and incubated for a further 15 min before performing mRNA purification with the RNeasy Mini Kit (Qiagen, Hilden, Germany) according to the manufacturer’s instructions. Subsequently, mRNA was dephosphorylated at 37 °C for 30 min with 15 U Antarctic phosphatase (New England Biolabs, Frankfurt am Main, Germany) and purified as described above. The concentration and purity of mRNAs were analyzed photometrically, and the size of the mRNAs was confirmed via 1% agarose gel electrophoresis and staining with 1x GelRed (Biotium, Fremont, CA, USA) in 1x Tris-borate-EDTA (TBE) buffer for 1 h at RT.

### 4.3. Cy3 Labeling of hGLuc mRNA

To evaluate the localization of the mRNA in the blood vessel walls, hGluc mRNA was labeled with the fluorophore Cy3. Therefore, Cy3 was linked to the mRNA using a copper-free click chemistry reaction. During IVT, 5-azido-C3-UTP (Jena Bioscience, Jena, Germany) molecules were integrated into the mRNA and subsequently labeled with Cy3. For this purpose, 25% 5-azido-C3-UTP (1.875 mM) and 75% Ψ (5.625 mM) were used for IVT as described in our previous study [53]. The azido mRNA was purified and coupled with DBCO-sulfo-Cy3 (Jena Bioscience) in a 5:1 ratio in a total volume of 40 µL nuclease-free water for 1 h at 37 °C. The labeled mRNA was purified using the RNeasy Mini Kit (Qiagen, Hilden, Germany) and evaluated via 1% agarose gel electrophoresis. 

### 4.4. Transfection of Cells with TIMP-1 mRNA 

During this stage, 3 × 10^5^ HUVECs, NuFFs, or EA.hy926 cells were seeded per well of a 6-well plate and cultivated overnight at 37 °C and 5% CO_2_ in the respective cell culture medium. Before the seeding of HUVECs, cell culture plates were coated with 0.1% gelatin in PBS for 15 min. The cells were transfected with 0.5, 1, or 1.5 µg synthetic mRNA complexed with Lipofectamine™ 2000 (L2000) in a 1:1 ratio in 1 mL Opti-MEM (except for 0.5 µg mRNA, where 1 µL L2000 was used). Opti-MEM alone (medium) or Opti-MEM with 1.5 µL L2000 (L2000) were used as controls. For the analysis of modified nucleotides on TIMP-1 mRNA expression efficiency, 1.5 µg of each modified TIMP-1 mRNA variant was complexed with 1.5 µL L2000 and transfected into EA.hy926 cells. Transfection mixtures were incubated for 20 min at RT. Before adding the transfection mixtures, the cells were washed once with DPBS, and the cells were incubated for 4 h at 37 °C and 5% CO_2_ with the transfection complexes. Then, the transfection mixtures were replaced with 2 mL of cell culture medium, and the cells were incubated for another 24 h at 37 °C and 5% CO_2_. Cell culture supernatants were collected, snap-frozen in liquid nitrogen, and stored at −80 °C until performing TIMP-1 protein-specific ELISA. Furthermore, the cells were lysed to detect intracellular TIMP-1 protein amounts via ELISA.

### 4.5. Lysis of Cells after TIMP-1 mRNA Transfection 

The cells were washed 2x with 1 mL cold DPBS, overlaid with 300 µL cold 1x RIPA buffer with 1:100 Halt™ Protease Inhibitor Cocktail (both from Thermo Fisher Scientific, Waltham, MA, USA), and incubated on ice for 5 min. The cells were then lysed via repeated pipetting, transferred to a reaction tube, and treated in an ultrasonic bath for 3 × 10 s with a 10 s pause in between. Subsequently, the cell lysates were centrifuged at 13,000 rpm, 4 °C for 25 min. The supernatant was collected, snap-frozen in liquid nitrogen, and stored at −80 °C until performing the TIMP-1 protein-specific ELISA. 

### 4.6. Application of Synthetic mRNA into Blood Vessel Walls 

#### 4.6.1. Tissue Preparation 

Porcine and human aortic tissues were transferred to 0.9% NaCl solution immediately after collection and used for microinjections on the same day. Before microinjection, the tissue was cut into 0.5 × 0.5 cm pieces and incubated for 30 min in an antibiotic solution composed of 250 mg/mL gentamicin (Sigma-Aldrich, St. Louis, MO, USA) and 1.25 mg/mL amphotericin B (PromoCell, Heidelberg, Germany) in DMEM followed by washing with DPBS (both from Thermo Fisher Scientific (Waltham, MA, USA)). 

#### 4.6.2. Injection of Synthetic hGLuc or TIMP-1 mRNA 

To analyze synthetic mRNA-mediated protein expression in the aortic vessel wall, synthetic mRNA was delivered ex vivo to porcine and human aortic vessel walls by microinjection from the side of the intima using MicronJet600™ hollow microneedles from NanoPass Technologies (Nes Ziona, Israel). Therefore, 1 µg of hGLuc mRNA or 3 or 5 µg TIMP-1 mRNA was complexed in a 1:1 ratio (µg:µL) with L2000 and incubated in a total volume of 50 µL Opti-MEM for 20 min at RT. The prepared mRNA solutions were drawn into a 1 mL syringe (Luer-LokTM, Becton, Dickinson and Company, Franklin Lakes, NJ, (USA)) using a cannula, which was replaced by the microinjection needle for the injection from the intraluminal side. After injection, samples were incubated for 5 min at RT, washed once in DPBS, and incubated at 37 °C in 5% CO_2_ in 1 mL EC medium in a 12-well plate for 24 to 48 h to analyze the luciferase activity, TIMP-1 amount by ELISA and the activity of exogenously produced TIMP-1 protein in the vessel wall using in situ zymography. Microinjection of 50 µL Opti-MEM and the corresponding amount of L2000 into the tissue served as controls. For the in-situ zymography, the tissue samples were incubated for 48 and 72 h at 4 °C in 10x zinc fixative (Formalin free, BD Pharmingen™, Becton, Dickinson and Company, Franklin Lakes, NJ, USA). 

To evaluate the localization of the mRNA after microinjection into the blood vessel wall, 1 µg Cy3 labeled hGLuc mRNA was complexed with 1 µL L2000 in 50 µL Opti-MEM for 20 min at RT. Tissue treated with Opti-MEM and the corresponding amount of L2000 served as the control. After the microinjection, the tissues were washed 1x in DPBS and fixed overnight in 4% paraformaldehyde (PFA) at 4 °C for histological analyses. 

#### 4.6.3. Detection of Luciferase Activity 

After the delivery of synthetic hGLuc mRNA into the aortic vessel wall, the expression of hGLuc in tissue supernatants was determined using a luciferase assay. Therefore, 40 µL of each supernatant was transferred in triplicate into a 96-well plate (Nunc Maxisorp, Thermo Fisher Scientific (Waltham, MA, USA)), 100 µL of 20 μg/mL coelenterazine (Carl Roth, Karlsruhe, Germany) in DPBS (w/o Ca^2+^/Mg^2+^) was injected automatically into each well, and luminescence was detected in Relative Light Units (RLU) using the microplate reader Mithras LB 940 (Berthold Technologies, Bad Wildbad, Germany). 

### 4.7. Histological Analysis 

#### 4.7.1. Detection of Microinjected mRNA in the Aortic Vessel Wall

After fixation, the samples were washed twice in 2 mL DPBS, transferred to embedding cassettes, and coated with 100% ethanol. Subsequently, automated dehydration and embedding in paraffin were performed by the Institute of Pathology at the University Hospital of Tübingen. Subsequently, tissue sections with a thickness of 2.5 µm were prepared. Afterward, the sections were deparaffinized 4× for 5 min in xylene rehydrated stepwise in an ethanol series: 2× 99% for 1 min, 2× 96% for 1 min, and 2× 70% for 1 min, washed twice in nuclease-free water, and then boiled for 2 min in TBE buffer (pH 9). The slides were cooled under running water, washed three times with DPBS, and, in the case of Cy3 mRNA injection, embedded in Fluoroshield Mounting Medium with DAPI (Vector Laboratories, Burlingame, CA, USA). Xylene and ethanol were both provided by AnalaR NORMAPUR (VWR, Darmstadt, Germany). To detect Cy3-labeled mRNA, fluorescence images were taken using an Axiovert135 microscope (Carl Zeiss, Oberkochen, Germany) and analyzed with AxioVision Rel 4.8 software. 

#### 4.7.2. In Situ Zymography

After fixation, the tissue was transferred to embedding cassettes, dehydrated, and embedded using a tissue embedding device and an automated tissue processor at the Institute of Pathology at the University Hospital of Tübingen. The tissue was then poured into paraffin blocks and 5 µm thick sections were prepared using the Microtome Microm HM 355S (Thermo Fisher Scientific (Waltham, MA, USA)) and mounted on SuperFrost^®^ microscope slides (R. Langenbrinck, Emmendingen, Germany). The tissue sections were deparaffinized in xylene (100% xylene, mixture of isomers, AnalaR NORMAPUR, VWR, Darmstadt, Germany) twice for 2 min and rehydrated using descending ethanol (AnalaR NORMAPUR, VWR, Darmstadt, Germany) series (100%, 80%, 70%, and 60%). The slides were washed in double-distilled water (ddH_2_O) and stained in a humidity chamber using an EnzChek™ Gelatinase/Collagenase Assay Kit (Thermo Fisher Scientific (Waltham, MA, USA)) by adding 250 µL substrate solution consisting of 1 mg/mL of the fluorescence-labeled DQ-gelatine diluted 1:50 in reaction buffer ((150 mM NaCl (NORMAPUR^®^, VWR International, LLC., Radnor, PA, USA), 5 mM CaCl_2_, 50 mM Tris-HCl, 0.2 mM sodium azide (all from Sigma-Aldrich, Darmstadt, Germany); pH 7.6)) to each section and incubation at 37 °C for 2 h. As a control, tissue sections were treated for 1 h with 20 mM EDTA to inhibit enzymatic metalloproteinase activity. These control slides were then incubated at 37 °C for 2 h with the substrate solution also containing 20 mM EDTA. Afterward, the sections were washed 3× for 1 min in ddH_2_O and fixed in 250 µL 4% PFA for 10 min in the dark, followed by 2× washing for 5 min in DPBS. Finally, the tissue sections were covered with Fluoroshield mounting medium containing DAPI (Vector Laboratories, Newark, NJ, USA) to stain the cell nuclei. Images were taken using the Axiovert135 fluorescence microscope (Carl Zeiss, Oberkochen, Germany) and analyzed using AxioVision Rel 4.8 program software. 

### 4.8. Human TIMP-1 ELISA

TIMP-1 amount was detected in the cell lysates and cell culture supernatants after TIMP-1 mRNA transfection and the microinjection of mRNA into the aortic tissue. The amount of TIMP-1 was detected using human TIMP-1 DuoSet ELISA (R&D Systems, Minneapolis, MN, USA) according to the manufacturer’s instructions. Cell supernatants and lysates were diluted (cell supernatants: 1:150–1:250; cell lysates 1:50) in 1% BSA in DPBS before ELISA. Supernatants of aortic vessel tissue cultivation were not diluted. The absorbance of the samples was measured using a microplate reader (Eon Synergy 2, BioTek Instruments, Winooski, VT, USA) at 450 nm with a correction wavelength of 540 nm. 

### 4.9. Statistical Analysis

The data are shown as the mean + standard deviation (SD) or standard error of the mean (SEM). One- or two-way analysis of variance (ANOVA) was performed followed by Tukey’s multiple comparisons test. All of the analyses were performed using Origin Pro 2024b (v10.15) and GraphPad Prism version 9.3.1. Differences of *p* < 0.05 were considered statistically significant.

## 5. Conclusions

In this study, the synthesis of TIMP-1 protein was induced by the exogenous delivery of synthetic TIMP-1 encoding mRNA into the aortic vessel to inhibit MMP-9. In vitro experiments demonstrated a significant increase in TIMP-1 protein expression in various cells following TIMP-1 mRNA transfection. Additionally, TIMP-1 protein expression was further increased through nucleotide modifications and the replacement of Ψ/m5C with me^1^Ψ. The functionality of the expressed protein was assessed using an appropriate ex vivo aortic vessel model, revealing a decrease in MMP-9 activity detected using in situ zymography 24 and 48 h after the microinjection of 5 µg TIMP-1 mRNA into the aortic vessel wall. These results indicate that administering TIMP-1 mRNA holds potential as a treatment strategy for aneurysms.

## Data Availability

The original contributions presented in the study are included in the article/Supplementary Material, further inquiries can be directed to the corresponding author.

## Supplementary Materials

The supporting information can be downloaded at: https://www.mdpi.com/article/10.3390/ijms25126599/s1.
