# Supplementary material for: A Novel Strategy for the Treatment of Aneurysms: Inhibition of MMP-9 Activity through the Delivery of TIMP-1 Encoding Synthetic mRNA into Arteries
Source: Int J Mol Sci. 2024 Jun 15;25(12):6599. doi: 10.3390/ijms25126599 (PMC11203431; doi:10.3390/ijms25126599)
Supplement: Supplementary file 1 [file ijms-25-06599-s001.zip › ijms-3025949-supplementary.pdf]

Supplementary Data

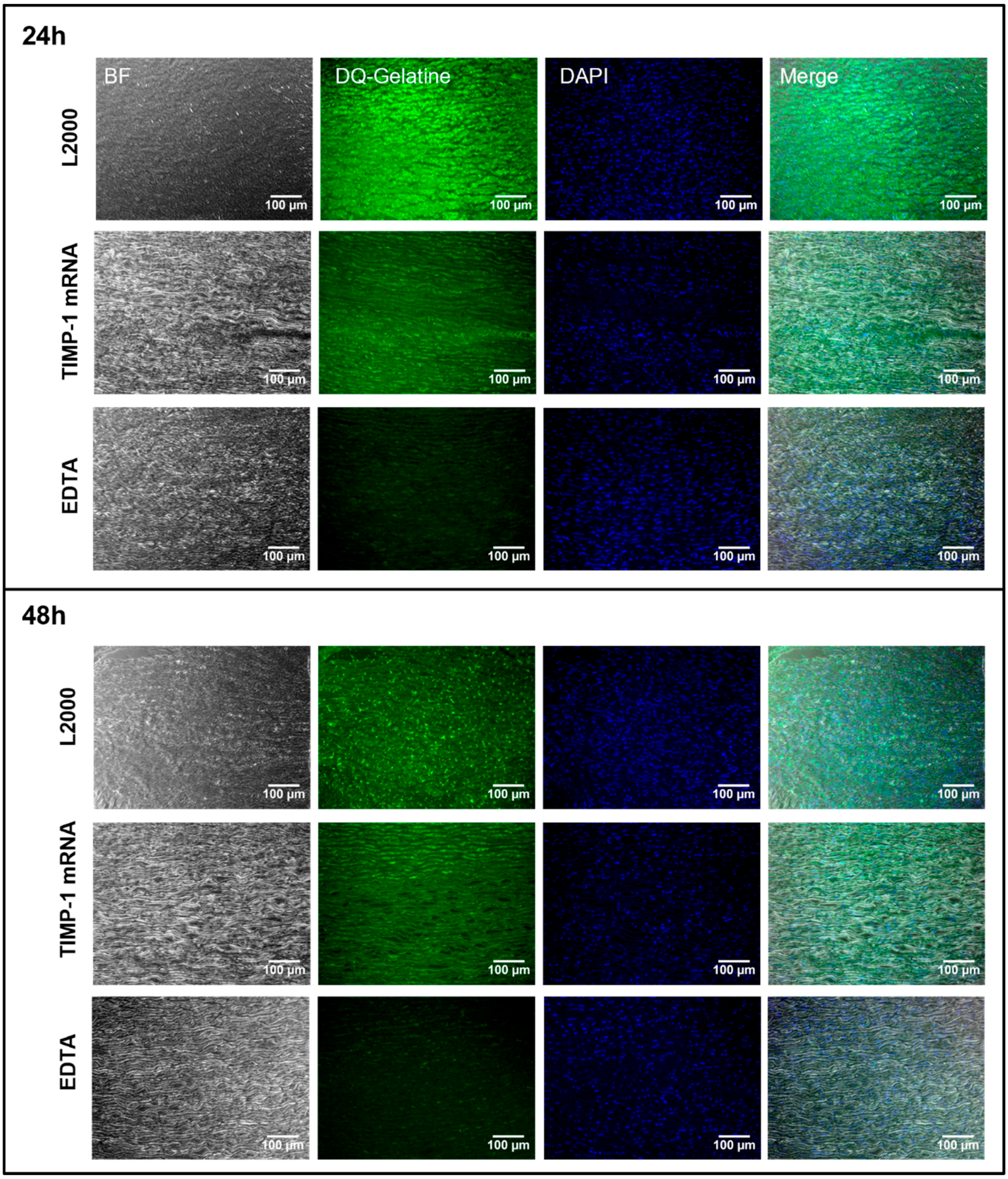

Figure S1: Analysis of MMP activity using in situ zymography in porcine aorta after microinjection of human TIMP-1 mRNA from the side of the adventitia. 5 µg TIMP-1 mRNA complexed 1:1 with L2000 in 50µl Opti-MEM were injected from the side of the adventitia and incubated for 24 and 48 h at 37°C and 5% CO_2. L2000 in Opti-MEM served as a control. Post incubation tissues were fixed, 5 µm paraffin sections were prepared and MMP-9 activity was visualized by using DQ-gelatin based in situ zymography. To verify enzymatic gelatin cleavage by MMPs, control sections were inhibited with EDTA before the addition of substrate. Cell nuclei were stained using a DAPI-containing mounting medium. DQ gelatin: green, Nuclei: blue. 20x magnification; (n=3).

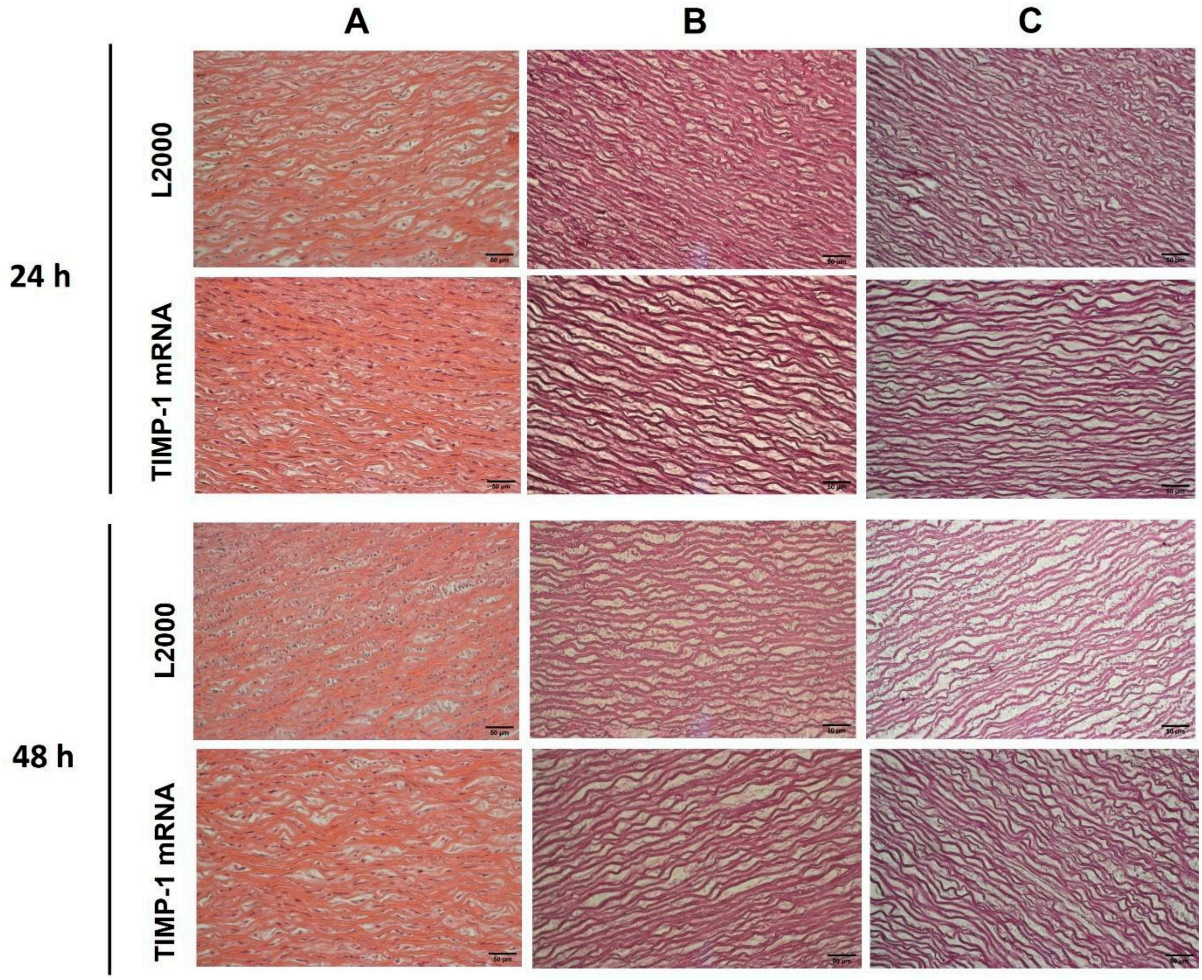

Figure S2: Histological staining of porcine aorta after microinjection of human TIMP-1 mRNA. 5 µg TIMP-1 mRNA complexed 1:1 with L2000 in 50 µl Opti-MEM were injected from the side of the intima and incubated for 24 and 48 h at 37°C and 5% CO_2. L2000 in Opti-MEM served as a control. Post incubation, the tissues were fixed, 5 µm paraffin sections were prepared and stained with A) Hematoxylin and Eosin (H&E), B) Elastica van Gieson, and C) Resorcin-Fuchsin to visualize cytoplasmic, nuclear, and extracellular matrix features including collagen and fibers. 10x magnification; (n=3).

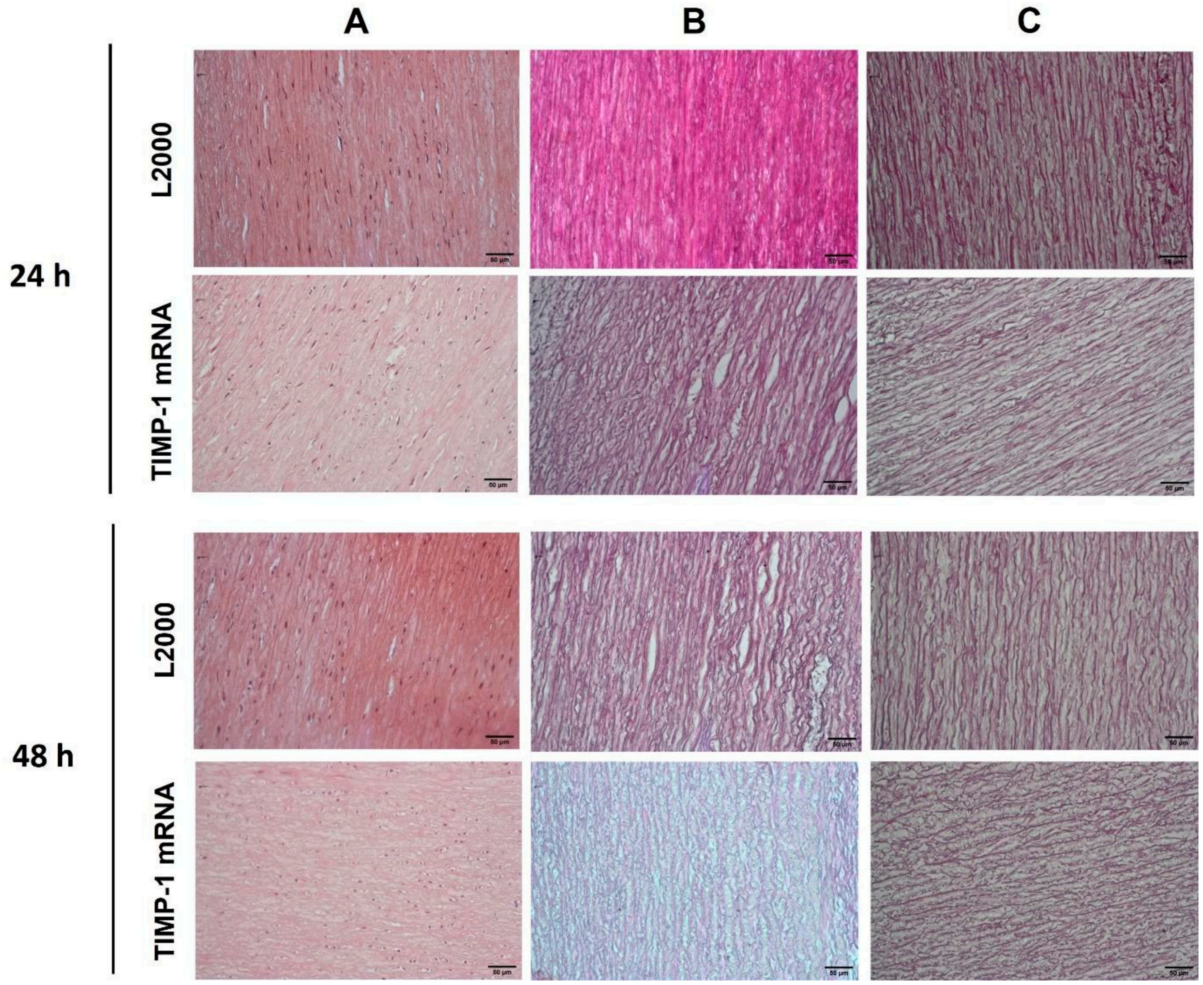

Figure S3: Histological staining of human aorta after microinjection of human TIMP-1 mRNA. 5 µg TIMP-1 mRNA complexed 1:1 with L2000 in 50 µl Opti-MEM were injected from the side of the intima and incubated for 24 and 48 h at 37°C and 5% CO_2. L2000 in Opti-MEM served as a control. Post incubation, the tissues were fixed, 5 µm paraffin sections were prepared and stained with A) Hematoxylin and Eosin (H&E), B) Elastica van Gieson, and C) Resorcin-Fuchsin to visualize cytoplasmic, nuclear, and extracellular matrix features including collagen and fibers. 10x magnification; (n=3).
